# Safe and dignified burial of a deceased from a highly contagious infectious disease ebolavirus: Socio-cultural and anthropological implications in the Eastern DR Congo

**DOI:** 10.1016/j.onehlt.2021.100309

**Published:** 2021-08-13

**Authors:** Franck Katembo Sikakulya, Michel Kalongo Ilumbulumbu, Sonia Fatuma Djuma, Gabriel Kambale Bunduki, Amos Kaghoma Sivulyamwenge, Malcolm K. Jones

**Affiliations:** a Faculty of Medicine, Université Catholique du Graben, Butembo, Democratic Republic of the Congo; b Faculty of Clinical Medicine and Dentistry, Department of Surgery, Kampala International University, Western Campus, Ishaka, Bushenyi, Uganda; c Epidemiological Surveillance, Beni, Democratic Republic of the Congo; d Malawi-Liverpool-Wellcome Trust Clinical Research Programme, Malawi; e School of Veterinary Sciences, The University of Queensland, Gatton, Australia

**Keywords:** Safe and dignified burial, Ebolavirus, Socio-cultural, Anthropological, Eastern DR Congo

## 1 Introduction

From 1976 to 2020, the Democratic Republic of Congo (DRC) registered 12 outbreaks of Zaire ebolavirus (EVD). The Eastern part of the country had recorded the tenth, largest and highly contagious EVD outbreak (from August 1, 2018 to June 21, 2020 in the Kivu) which ended with 3317 confirmed cases and a total of 2287 deaths, with a case fatality rate of 68.9% [1]. The twelfth EVD outbreak was declared in Butembo on 7th February 2021 by the DRC Ministry of Health, and was declared as ended by the same organization on 3rd May 2021. This outbreak had a total of 12 cases, 6 deaths (case fatality rate 50%) [2].

The DRC, with the help of the World Health Organization (WHO) and a number of partners (regional and international), battled the tenth outbreak EVD outbreak by implementing a range of response plans. Among these were the practice of safe and dignified burials and a bundle of interventions (including infection prevention and control practices, case management, surveillance and contact tracing, community sensitization, good laboratory services, etc.) [3]. Only a few cases of EVD were nosocomial, while the transmission of most reported cases in the community were by taking care of the sick or by handling corpses during traditional funerals. Promotion of these practices raised community resistance [3,4]. This resistance was due to misunderstandings of public health practices such as isolation and safe burials. These safe practices were perceived as separation with the beloved deceased, denial of human touch at the point of death, and replacement of traditional funeral rites by safe burials while these rites are considered of high importance in the cultural and social context of the region. Furthermore, the presence of armed conflict in the zone led to a number of attacks on EVD response teams and facilities; resulting in release of infected cases in the community and contact with contaminated instruments and bodies of Ebola victims during unsafe traditional funeral ceremonies. This has impacted negatively on EVD response plans to tackle the Outbreak in North Kivu [5].

Given the extent to which human behavior influences how infectious diseases spread, the relevance of social sciences in responding to health crises is well known. The West Africa EVD outbreak, between 2014 and 2016, highlighted the importance of anthropologists assisting medical stakeholders in the acceptance of safe and dignified burials by the community, which was one of the key components of Sierra Leone's efforts to end the EVD [6]. Information on socio-cultural and anthropological implications on safe and dignified burial of a deceased from a EVD in North-Kivu is limited. Therefore, this editorial seeks to highlight some insight into how gathering limitation for safe and dignified burial was accepted by the community during the tenth EVD outbreak.

## 2 Community beliefs on death and burial ceremony in North Kivu

North Kivu is a province in North-Eastern part of DRC between Maniema province to the west, Province Orientale to the north and northwest, South Kivu to the south and bordering Rwanda and Uganda to the east. The province has an estimated population of 6 million people (~39 people/km^2^). Of these, 60% live in rural areas. Due to its varied ethnic mix, porous borders with Rwanda and Uganda, and the continued presence of foreign and Congolese armed organizations, North Kivu is one of the most volatile regions in the country [4]. Although the ethnic composition of the province is complex, the cultural of burial ceremony remain the same among different people groups.

Once a person is dead, the body is taken to the family's patriarch home. It is not usual to leave a body at the hospital. There is usually a one-night vigil before the burial, and it is expected that the body will remain in the home during this period. Further, upon pronouncement of death, the information will be delivered to a responsible dependant who must deliver the most plausible cause of death. The information from the doctors is usually trusted by the family members, although some families still consider metaphysical explanations for the death, such as death as a result of curse or divine or ancestor disfavour, or a combination of both medical and metaphysical.

Once the body is delivered to the family home, most families still wash the body, a task performed by certain family representatives. Men will wash a man's body, and women will wash a woman's body. After washing, the body will be sprayed with perfume and dressed in good clothes and placed in a coffin prior to burial. In villages and rural areas, however, coffins are less readily available, and often the body is wrapped in cloth. Islamic communities also wrap the body of their deceased in cloth but do not use a coffin. The cloth that is used to carry the body to the burial site (as opposed to the cloth that the body is wrapped in) is often retained by the family. The night before the burial, the friends and members of the family will gather around the body to give their last respects to the deceased. Crying, eating, drinking will characterize the ceremony overnight. Some members will even touch the cloths and body of the deceased to express their grief. Most families prefer to bury the body of the deceased in their ancestral home but due to the arm conflict ongoing in the region, the burial ceremonies are conducted in specific places in the city or village where all the clan, family and friends will attend the ceremony.

From the ceremony, the participants will gather at home of the deceased for a family meal to conclude the ceremony of the deceased. In the region, there is a belief that if burial is not conducted well, or to the standards prescribed by the community, the deceased has not been given the opportunity to rest in peace, and their life (and life in general) is disrespected. Failure to perform a “proper” burial can have serious consequences for the family and the society.

## 3 The actions of anthropologists and community benefit in stopping EVD outbreak

When there is an outbreak, the dignified burial ceremony should stick as closely as possible to local burial and funeral customs, modifying or altering only those that are medically unsafe and this should be done by burial trained specialists otherwise, threats from dangerous diseases can induce fear and panic, and governments may respond to political and popular pressure by enacting restrictions that unjustly restrict personal freedoms [7].

Local actors are involved in this negotiation process, formulating locally-appropriate practices concerning death, mourning and burial. Local practices do not remain constant, but rather alter and adapt in response to changing circumstances. Communities in the North Kivu are pragmatic and it has been reported that both individual and collective behaviours have been adjusted in the context of the Ebola outbreak [8]. Communities are best positioned to offer appropriate changes to local practices when they understand the risk of transmission involved with preparing a body after death and for burial.

Local discussion and agreement on how the safe and dignified burial (SDB) was conducted and some categories of local leaders (Religious and community leaders) were selected to attend the meeting. From this meeting, their views were incorporated and sensitization of local community about the outbreak and burial ceremony and modifications aligned with SDB guidelines. A team of local leaders was incorporated alongside formal Safe and dignified Burial teams to conduct burial ceremonies.

For coordination with religious leaders in North Kivu, it was recommended that the protestant church platform and its youth section be directly engaged, in addition to Catholic and Muslim leaders, in forming responses. In more remote areas, beyond immediate urban centres, customary authorities were engaged in formulating locally appropriate responses. Seeing the dead body is a significant component of local practices following a death. During the outbreak, local community adhered to SDB and suggested that preliminary engagement with local residents and counselling staff suggest that trusted family members to be allowed to access and safely view the body without physical contact, or to view the body through a body bag as this would allow them to see the form of the body without being at risk of exposure. This, however, requires further investigation.

In urban areas such as Beni and Butembo, the practice of washing the body is not widespread and the change to keep the body in the mortuary and use of perfumes to spray was more acceptable. SDB protocols was adjusted in the way to allow family members to spray the body with perfume and thereby participate in the preparation of the body in a medically-safe way. If this level of access is not feasible, then families were asked to select the perfume for the medical or SDB team to spray on the body as it is prepared for burial, and if possible, selected family members were suggested to view this act, potentially with their religious leader present to offer a prayer or blessing. The family members were requested to provide appropriate clothes for the SDB or medical team to dress the body prior to burial as this practice is still considered good behavior in the region.

Timely information about the patient and their medical progress during admission was suggested by the local community to adhere to the plan and strategies to control the spread of the disease within the community. The family members of the patients should be informed in time about the patient and if death occur, they should be informed before the preparation of burial ceremony so that they can participate as mentioned above.

Local communities suggested that the SDB team should not be composed of only unknown people, but also local members were suggested to be trained and be included and participate to the SDB can act as a liaison between a family and the burial crew, even though they are not actively involved in making the body medically safe. The importance of actively engaging local community members into burial teams and promoting successful community engagement has been widely documented in prior epidemics in the DRC, Uganda, and West Africa [5,9,10].

Psychological support to patients and members of the family affected by the EVD was a key element to control the outbreak by doing a continuous psychological support. During this support, family members and patients were sensitized about the disease and the preventive measures to avoid its spread. Local psychologists, updated by the experts from EVD, were working together to carry this task. A clear explanation about the SDB procedures agreed at the local level was being carefully explained to all community members, and opportunity provided for them to ask questions during the sensitization period of the local community. Such engagement reduced the risks of surprise, erroneous assumptions, and suspicions, all of which contributed to reluctance, refusal, and resistance.

## 4 Implications of Anthropologists in multi-sector approaches to infectious disease control

Anthropologists have always been engaged in research on human health and illness, diagnosis and treatment, and death and dying. Anthropological research into infectious disease has frequently focused on the characteristics of illness: cultural views of disease entities, understandings of aetiology, diagnostic categories, and treatment-seeking behaviours [11].

Pathogens are intertwined with the social world of humans. From HIV to malaria, from Lyme disease to tuberculosis, Ebola to COVID-19, infectious diseases understanding of the biocultural approach has been emphasized by the fact that they cannot be understood solely on the basis of biology, but must be understood in the cultural and social context in which they exist [12].

To tackle an outbreak or any infectious disease in a region, the connections between biological, political, economic, socio-cultural, and environmental elements, as well as how these elements affect pathogen emergence, prevention, treatment, dissemination, cultural experiences, and global impact, must be understood by decision-makers [4]. In the context of EVD outbreak in the eastern part of the DRC, it was shown that the impact of anthropologists was remarkable to control the spread of the disease by understanding the disease and sensitize the local community about safe and dignify burial ceremony, a key element in infectious disease control. During the EVD in Eastern DRC as in the past outbreak in Western Africa [6], the anthropologists involved local leaders (religious and customary authorities) to sensitize the community about the infectious disease control. Despite the fact that anthropologists have been involved in disease outbreaks for years, their position in emergencies is likely to grow as more people advocate for deeper integration of sociocultural approaches to health crises. As a matter of fact, the community of emergency responders must be proactive in developing a multidisciplinary strategy to public health emergencies [13]. Contagion, fear, and stigma all have a significant impact on the disease experience, access to care, and, as a result, disease progression and outcomes. Political and economic issues, such as poverty and war, worsen infectious diseases. Because a fruitful way of comprehending the human condition is to comprehend the connections between diseases, culture, social inequity, and ecology.

## 5 Conclusion

Death, burial, funeral rites, and mourning beliefs and traditions can have a direct impact on Ebola transmission and influence trust between communities and responders. In the context of EVD in North Kivu, two-way dialogue and community consultations ensured community members understand the need for SDB and to raise awareness about the use of locally appropriate SDB. Rumours about the care of the deceased and the intentions of the burial teams were also reduced thanks to a well-managed and open process.

## Ethical approval and consent to participate

Not applicable.

## Consent for publication

Not applicable.

## Competing interest

Authors declare no competing interest.

## Author's contributions

All authors contributed equally, read and approved the final manuscript.

## Funding

The authors GKB and FKS are funded by the Malawi-Liverpool-Wellcome Trust (MLW) core funding (Wellcome Trust Core grant 206545/Z/17/Z) and by Safe Surgery in War Zone DRC (SSWZ-DRC) respectively. The funders had no role in study design, decision to publish, or preparation of this manuscript.
